# Association of Apolipoprotein A5 Gene −1131T>C Polymorphism with the Risk of Metabolic Syndrome in Korean Subjects

**DOI:** 10.1155/2013/585134

**Published:** 2013-01-28

**Authors:** Kwang Hoon Song, Seongwon Cha, Sung-Gon Yu, Hyunjoo Yu, Soo A. Oh, Nam-Sik Kang

**Affiliations:** Division of Constitutional Medicine Research, and Herbal Medicine Research Division, Korea Institute of Oriental Medicine (KIOM), 483 Expo-ro, Yuseong-gu, Daejeon 305-811, Republic of Korea

## Abstract

We assessed the associations between the *APOA5*  −1131T>C polymorphism and lipid parameters and other risk factors of the metabolic syndrome in Korean subjects. A total of 2,901 participants from 20 oriental medical hospitals in Korea were enrolled between 2006 and 2011. According to the modified National Cholesterol Education Program Adult Treatment Panel III definitions, subjects were classified into the metabolic syndrome group and control group. The *APOA5*  −1131T>C genotype was significantly associated with serum high-density lipoprotein cholesterol levels (effect = − 1.700 mg/dL, *P=6.550*-E07) in the total study population after adjustment for differences in age and gender. The association of the *APOA5*  −1131T>C genotype with serum log-transformed triglyceride was also significant in an additive genetic model (effect = 0.056 mg/dL, *P=2.286*E-19). After adjustment for age and gender, we determined that the odds ratio for the occurrence of the metabolic syndrome was 1.322 for C-allele carriers in the additive model (95% CI = [1.165 − 1.501], *P=1.48*E-05). In the current study, we demonstrated that the *APOA5*  −1131T>C polymorphism is associated with the metabolic syndrome because of its remarkable effect on serum triglyceride levels in Korean subjects.

## 1. Introduction

Metabolic syndrome (MS) is defined by the clustering of cardiovascular risk factors such as abdominal obesity, dyslipidemia, hypertension, and hyperglycemia and is significantly associated with increased risk of developing diabetes and cardiovascular disease [1, 2]. The prevalence of this syndrome has significantly increased in Koreans in the past decade and affected an estimated 31.3% of the population in 2007 [3]. Susceptibility to MS is influenced by the imbalance between caloric intake and expenditure, and by genetic factors [4–6]. Moreover, genome-wide scans have revealed various chromosomal regions with suggestive linkage to MS [7–9]. Recent Genome-Wide Association Study (GWAS) results from seven studies participating in the single nucleotide polymorphisms Typing for Association with Multiple Phenotypes from Existing Epidemiologic Data Consortium revealed that genetic effects on lipid levels were pronounced and that genes with variants influencing MS per se included LPL, CETP, and the APOA-cluster (*APOA5*, *ZNF259*, and *BUD13*), which are known to play an important role in lipid metabolism [10].

The gene encoding a novel member of the apolipoprotein family, human apolipoprotein A5 (*APOA5*), is located on chromosome 11q23 close to the *APOA1–C3–A4* gene cluster. *APOA5* comprises 4 exons encoding 366 amino acids and is an important determinant of plasma triglyceride (TG) levels, a major risk factor for coronary artery disease [11]. Genetic polymorphisms of *APOA5* are associated with hypertriglyceridemia [11–13]. One of these *APOA5* polymorphisms, −1131T>C (rs662799), which is located in the *APOA5* promoter region, has been associated with elevated plasma TG levels and reduced plasma high-density lipoprotein cholesterol (HDL-C) levels [14–17].

Several studies indicated an ethnic-specific association of the *APOA5* −1131T>C polymorphism with MS [18–22]. Therefore, we assessed the associations between *APOA5* −1131T>C polymorphism and lipid parameters and other risk factors of MS in Korean subjects.

## 2. Materials and Methods

### 2.1. Study Population

Data were retrospectively collected from 20 oriental medical hospitals in Korea between 2006 and 2011. The current study population comprised 2,901 participants. Recruited participants were outpatients who had at least 4 visits. To avoid potential confounding of the association between the *APOA5* −1131T>C polymorphism and preexisting diseases subjects with a history of cardiovascular disease, hypertension, cancer, liver disease and diabetes (as recorded on case report forms) were excluded. All samples and clinical information were deposited in the databank of Sasang Constitutional Medicine (DB-SCM), which was renamed to Korea Constitution Multicenter Study (KCMS) recently, in the Korea Institute of Oriental Medicine as previously described [23]. All subjects provided written informed consent, and this study was approved by the Institutional Review Board of the Korea Institute of Oriental Medicine.

### 2.2. Genotyping of Single Nucleotide Polymorphism

The genotype of the *APOA5* −1131 polymorphism was determined using an unlabeled oligonucleotide probe (UOP) that spans the polymorphic nucleotide [24]. The UOP was designed from a perfectly matched duplex with the T allele of *APOP5* −1131. Briefly, small segments of genomic sequence spanning the *APOA5* polymorphic site were amplified by polymerase chain reaction (PCR) using 50 ng of genomic DNAs as a template and a set of primers (*APOA5* −1131-F1 ACTCTGAGCCCCAGGAAC, *APOA5 *−1131-R1 GAGTGGAGTTCAGCTTTTCC). PCR amplification was performed via a heating cycle (50°C for 2 min and 95°C for 10 min), followed by an amplifying step (40 cycles of 95°C for 20 seconds, 56°C for 20 seconds, and 72°C for 20 seconds) in a model C1000 thermal cycler (Bio-Rad). The PCR product was diluted with 2 volumes of distilled water, and the diluent was mixed with 6 volumes of probe solution containing 1 *μ*M UOP, 5 *μ*M Syto 9 (Invitrogen), 12.5 mM EDTA, and 10 mM Tris (pH 8.0) and subsequently subjected to a thermal reaction for genotyping using a Lightcycler 2.0 and 480II instruments (Roche Diagnostics). The thermal reaction consisted of a denaturation step at 95°C for 5 seconds, an annealing step at 60°C for 1 min to allow annealing of complementary strands (UOP, *APOA5* −1131-S AGCTTTTCCTCATGGGGCAAATCTCACTT), and a melting step with a gradual increase in temperature at a rate of 0.1°C/second until 95°C, when fluorescence emissions were read. The genotype of each PCR product was then determined from 3 melting patterns (major homozygote, heterozygote, and minor homozygote) on the basis of the temperature where the corresponding UOP melted.

### 2.3. Criteria for MS

MS was defined according to the National Cholesterol Education Program Adult Treatment Panel III (NECP ATP III) guidelines, which stipulated that at least 3 of the following 5 criteria had to be met: (1) fasting blood glucose (FBG) ≥ 100 mg/dL, (2) TG ≥ 150 mg/dL, (3) HDL-C ≤ 40 mg/dL for males and 50 mg/dL for females, (4) systolic blood pressure (SBP) ≥ 130 mm Hg and/or diastolic blood pressure (DBP) ≥ 85 mm Hg, and (5) central obesity with waist circumference (WC) ≥ 90 cm for males and ≥80 cm for females. For FBG levels, we referred to the American Diabetes Association (ADA) guidelines [25] and used a modified WC cut-off of ≥90 cm in males and ≥80 cm in females as abdominal obesity cutoff points [26]. 

### 2.4. Statistics

The quantitative variables were presented as mean ± standard deviation. The allele frequency of the *APOA5* −1131T>C polymorphism was determined by gene counting. Chi-square analysis was used to evaluate whether the *APOA5* −1131T>C polymorphism was in Hardy-Weinberg equilibrium in the population. For comparison of quantitative variables between subjects with MS and controls, MS risk factors (SBP, DBP, TG, WC, HDL-C, and FBG), we used Student's *t*-test. Multiple linear regression analysis was used to identify significant variables affected by the *APOA5* genotype, and effect sizes (slope) were presented as changes in minor allele carrier in an additive model, adjusting for the effects of age and gender. Tests for difference of proportions were conducted to evaluate the significance of genotype subgroup proportion differences in MS criteria. To identify the association with a binary variable, for example, MS, we used logistic regression analysis. Multiple logistic regression analyses were conducted to calculate the odds ratio (OR) of the risk of MS for the carriers of the minor allele of the *APOA5* −1131T>C polymorphism in an additive model, adjusting the effects of age and/or gender [27]. The effect sizes (slope and OR) were estimated with their respective 95% confidence interval (CI). A *P-*value of < 0.05 was considered statistically significant. All statistical analyses were performed using SPSS (SPSS Inc., Chicago, IL,USA; V14.0 for Windows) and R (version 2.12.0).

## 3. Results

### 3.1. Characteristics and Prevalence of MS in the Total Study Population

A total of 2,901 individuals (1,040 males and 1,861 females) were enrolled in this study. Major clinical parameters including age, SBP, DBP, WC, FBG, log transformed TG (LogTG), and HDL-C levels of subjects with MS and controls in the total study population and according to gender are summarized in Table 1 and Supplementary Table 1 available online at http://dx.doi.org/10.1155/2013/585134. Using the modified NECP ATP III diagnostic criteria, we detected a 34.61% prevalence of MS in the study population (33.85% in males and 35.03% in females). The genotype distribution of the *APOA5 −*1131T>C polymorphism among the 2,901 subjects analyzed was as follows: 1,404 (48.40%) subjects were homozygous for the T allele (TT), 1,237 (42.64%) subjects were heterozygous (TC), and 260 (8.96%) subjects were homozygous for the C allele (CC). The minor allele (C) frequency of the *APOA5 −*1131T>C polymorphism was 0.302, and the genotype distributions did not deviate from Hardy-Weinberg equilibrium (*P* > 0.05).

### 3.2. Association of the *APOA5 −*1131T>C Genotype with Clinical Parameters of MS

To address the association and impact of the *APOA5 −*1131T>C polymorphism on clinical parameters of MS, we evaluated the mean values of age, SBP, DBP, WC, FBG, TG, and HDL-C of each genotype and performed multiple regression analysis using the total study population, subjects with MS, and controls. There was a significant association between serum HDL-C levels and the *APOA5 −*1131T>C genotype (effect = −1.700 mg/dL, *P* = 6.550E-07) in the total population after adjustment for differences in age and gender. The association of *APOA5 −*1131T>C with serum LogTG values was also significant in an additive genetic model. The TC genotype group showed higher serum LogTG levels (2.04 ± 0.24 mg/dL) than the TT genotype group (2.00 ± 0.23 mg/dL), and the CC genotype group showed the highest serum LogTG levels (2.13 ± 0.24 mg/dL). However, there were no significant differences in SBP, DBP, WC, and FBG between these genotype groups (Table 2). The C allele was associated with higher levels of LogTG (MS: effect = 0.046 mg/dL, *P* = 1.457E-06; control: effect = 0.042 mg/dL, *P* = 2.158E-10), and the minor allele had a lowering effect on the levels of HDL-C levels (MS: effect = −0.818 mg/dL, *P* = 0.032; control: effect = −1.304 mg/dL, *P* = 0.003). 

Since it has been reported that gender has an important impact on the correlation of *APOA5* with serum HDL-C and TG levels [22, 23], we also analyzed the data according to gender (Supplementary Table 2). In both male and female subjects, the C allele correlated with higher levels of TG (in male, effect = 0.061 mg/dL, *P* = 1.963E-08; in female, effect = 0.053 mg/dL, *P* = 3.772E-12) and with lower HDL-C levels (in male, effect = −2.102 mg/dL, *P* = 3.810E-05; in female, effect = −1.444 mg/dL, *P* = 0.001) in the total population. Furthermore, the minor allele had lowering effects on HDL-C levels only for the male controls (effect = −2.092 mg/dL, *P* = 0.001).

### 3.3. Prevalence and Odds Ratio of MS according to Carriers of the Minor Allele of the* APOA5 −*1131T>C Polymorphism

The relative frequencies of the individual risk factors of the MS in the total population and according to gender are summarized in Table 3. The prevalence of MS was 32.19% (31.86% in males and 32.37% in females), 34.68% (33.33% in males and 35.48% in females), and 47.31% (45.19% in males and 48.72% in females) in subjects with the TT, TC, and CC genotype, respectively. Low HDL-C levels and large WC were the most common risk factor of MS regardless of the genotype in the total population. A high TG level was ranked the lowest priority in the frequencies of individual risk factors of MS in subjects with the TT and TC genotypes but it was significantly more prevalent in subjects with the CC genotype. Interestingly, there was a high frequency of the minor allele (C) of the *APOA5 −*1131 polymorphism in subjects with MS (control, 0.285 versus MS, 0.336). Moreover, the minor allele (C) of the *APOA5 −*1131 polymorphism in subjects with MS was more prevalent in females (control, 0.273 versus MS, 0.327) than in males (control, 0.307 versus MS, 0.352) (Table 4). 

Since our findings indicated that the increase in the minor allele (C) frequency of the* APOA5 −*1131 polymorphism was correlated significantly with the prevalence of MS, we calculated ORs for MS according to the carriers of the minor allele of the polymorphism using logistic regression analysis in an additive model (Table 4). After adjustment for age and gender, we determined that the OR for the occurrence of MS was 1.322 for C-allele carriers (95% CI = [1.165–1.501], *P* = 1.48*E* − 05). In addition, the association between the C allele of the *APOA5* polymorphism and MS was stable in both males (OR = 1.285, 95% CI = [1.050–1.574], *P* = 0.015) and females (OR = 1.339, 95% CI = [1.138–1.576], *P* = 4.52*E* − 04) after adjustment for age.

## 4. Discussion

Naturally occurring polymorphisms of *APOA5* are associated with hypertriglyceridemia, which is a component of MS [6, 11, 18, 23]. Among the *APOA5* polymorphisms, −1131T>C is a regulatory variant of *APOA5* that is predominantly associated with TG levels; a recent study documented the strong association of the −1131T>C polymorphism with serum TG levels in a dose-dependent manner [11, 28]. In addition, a significant dose-dependent association has been reported between the *APOA5 *−1131T>C polymorphism and the risk of coronary heart disease in an analogous dose-dependent manner, with about an 18% higher risk per C allele, providing evidence for a causal association between TG-mediated pathways and coronary heart disease [28]. 

In this study, we investigated the roles of the *APOA5 −*1131T>C polymorphism in serum lipid levels and the association of the prevalence of MS in the Korean population. The *APOA5 −*1131T>C polymorphism was found to be significantly associated with serum TG and HDL-C levels (Table 2, Supplementary Table 2), reemphasizing that it has a significant impact on both serum HDL-C and TG levels. Low HDL-C levels are associated with high blood TG levels. It has been suggested that deficiency of *APOA5* delays TG hydrolysis and reduces the availability of surface components of TG-rich lipoproteins, which contribute to HDL-C formation, thereby leading to decreased formation of HDL-C [17, 29]. Since the *APOA5 −*1131T>C polymorphism is located in the promoter region of *APOA5*, it may alter the *APOA5* expression and impair its function, and cooperative effects between *APOA5* polymorphisms cannot be excluded. Moreover, results from *in vitro* studies suggested that haplotypes carrying rare alleles of *APOA5* were associated with about 50% lower gene expression than wild-type haplotype [28].

The minor allele (C) frequency of *APOA5 −*1131 of 0.302 in our study population was similar to the frequency previously reported in other Korean subjects [30, 31]. However, we found that there was a significant difference in the frequency of the minor allele (C) of the *APOA5 −*1131 polymorphism in subjects with MS. Analysis of the overall sample indicated that the mean value in subjects with the *APOA5 −*1131CC genotype (164.42 ± 126.22 mg/dL) met the MS criteria for high TG, while subjects with the TT and TC genotypes did not (114.58 ± 66.55 and 128.97 ± 82.54 mg/dL, respectively). The differences in the prevalence of MS according to the *APOA5 −*1131 genotype suggest that the increased frequency of the minor allele of the *APOA5 −*1131T>C polymorphism may function as a significant risk factor of MS susceptibility. Furthermore, it should be noted that we observed that subjects with the *APOA5 −*1131CC genotype showed higher ranking of the “high TG” parameter compared to subjects with the other genotypes, suggesting that the C-allele (and in particular the CC genotype) could be a risk factor for MS. 

Gender-specific influences may interact with the *APOA5* −1131T>C polymorphism to modulate TG and HDL-C levels; (1) C carriers had a higher TG levels and lower HDL-C levels in males than females, (2) the effect of the *APOA5* −1131T>C polymorphism on TG levels was pronounced in the MS group than in the control group of males, and (3) a lower HDL-C levels was only significant in the control group of males.

The C allele of the *APOA5 −*1131 polymorphism was significantly associated with an increased risk of MS in a multiple logistic regression analysis. In agreement with previous reports, we also found that the susceptibility to develop MS was higher in subjects with the *APOA5 −*1131CC genotype than in those with the TT and TC genotypes, which is indicative of an effect of this polymorphism on serum TG levels. The present study not only discloses the exceptionally higher prevalence of MS in *APOA5 −*1131CC carriers than in TC and CC carriers, but also suggests that identification of carriers of the C allele of the *APOA5* −1131 polymorphism may be helpful for preventive care for MS. In addition, the percentage of participants with one or more MS risk factors suggests that subjects harboring the *APOA5 −*1131CC genotype are potentially at risk for developing MS (Supplementary Figure 1).

## 5. Conclusions

In conclusion, the present study describes that the *APOA5 −*1131T>C polymorphism is significantly associated with serum HDL-C and TG levels, as well as with MS risk, because of its pronounced effect on serum TG levels in Korean subjects. Therefore, we believe that the identification of carriers of the minor allele of the *APOA5 −*1131T>C polymorphism may prove helpful in predicting MS susceptibility and overall risk assessment for metabolic diseases such as cardiovascular disease. Additional studies, including not only other sequence variants of *APOA5* but also a large number of uninvestigated single nucleotide polymorphism-MS associations via genome-wide association studies, will further enhance the validity of associations and the causative relationships between the genetic variants and MS.

## Supplementary Material

Supplementary Table 1. General characteristics of the study subjects according to gender.
Supplementary Table 2. Association of the APOA5 -1131T>C polymorphism with MS parameters according to gender.

## Figures and Tables

**Table 1:** General characteristics of the study subjects.

Variables	Total (2901)	MS (1004)	Control (1897)	*P* value
Age (years)	47.76 ± 15.46	55.45 ± 13.66	43.69 ± 14.79	1.198**E** − 92
SBP (mm Hg)	119.91 ± 15.66	128.94 ± 15.55	115.13 ± 13.47	3.932**E** − 109
DBP (mm Hg)	77.04 ± 11.16	82.66 ± 11.08	74.07 ± 10.01	1.446**E** − 84
WC (cm)	83.91 ± 9.76	90.88 ± 7.73	80.21 ± 8.65	3.990**E** − 204
FBG (mg/dL)	99.05 ± 27.42	111.75 ± 36.62	92.33 ± 17.62	9.339**E** − 52
LogTG (mg/dL)	2.03 ± 0.24	1.94 ± 0.19	2.20 ± 0.21	1.991**E** − 192
HDL-C (mg/dL)	47.26 ± 12.57	39.78 ± 8.61	51.22 ± 12.53	1.617**E** − 160

Values are indicated as the mean ± standard deviation.

*P* value: Student's *t*-test result between MS and Control.

Abbreviations: MS: metabolic syndrome; SBP: systolic blood pressure; DBP: diastolic blood pressure; WC: waist circumference; FBG: fasting blood glucose; LogTG: log-transformed triglyceride; HDL-C: high-density lipoprotein cholesterol.

Bold indicates statistical significance (*P* < 0.05).

**Table 2:** Association of the *APOA5* −1131T>C polymorphism with MS parameters in the study population.

	Variables	TT/TC/CC	Slope (95% CI)	*P* value
Total (2901)	Number (%)	1404 (48.40)/1237 (42.64)/260 (8.96)	—	—
	Age (years)	47.93 ± 15.54/47.33 ± 15.53/48.92 ± 14.61	—	—
	SBP (mm Hg)	119.67 ± 15.77/119.69 ± 15.58/122.27 ± 15.33	0.589 (−0.244, 1.422)	0.166
	DBP (mm Hg)	76.88 ± 11.37/76.99 ± 11.13/78.20 ± 10.10	0.326 (−0.282, 0.934)	0.264
	WC (cm)	83.94 ± 9.72/83.65 ± 9.81/84.97 ± 9.73	0.051 (−0.451, 0.553)	0.843
	FBG (mg/dL)	99.02 ± 27.91/98.98 ± 26.24/99.61 ± 30.27	−0.013 (−1.505, 1.479)	0.987
	LogTG (mg/dL)	2.00 ± 0.23/2.04 ± 0.24/2.13 ± 0.24	0.056 (0.044, 0.069)	2.286**E** − 19
	HDL-C (mg/dL)	48.34 ± 12.84/46.69 ± 12.43/44.16 ± 10.97	−1.700 (−2.368, −1.031)	6.550**E** − 07

MS (1004)	Number (%)	452 (15.58)/429 (14.79)/123 (4.24)	—	—
	Age (years)	55.89 ± 13.95/54.92 ± 13.68/55.71 ± 12.48	—	—
	SBP (mm Hg)	129.38 ± 16.09/127.77 ± 15.48/131.40 ± 13.39	0.185 (−1.218, 1.587)	0.796
	DBP (mm Hg)	82.73 ± 11.38/82.40 ± 11.23/83.30 ± 9.33	0.065 (−0.941, 1.071)	0.899
	WC (cm)	91.17 ± 7.51/90.57 ± 8.00/90.92 ± 7.56	−0.328 (−1.018, 0.362)	0.351
	FBG (mg/dL)	112.38 ± 37.56/111.68 ± 34.69/109.70 ± 39.73	1.103 (−4.389, 2.182)	0.510
	LogTG (mg/dL)	2.17 ± 0.20/2.22 ± 0.22/2.27 ± 0.24	0.046 (0.028, 0.065)	1.457**E** − 06
	HDL-C (mg/dL)	40.62 ± 8.94/39.05 ± 8.45/39.28 ± 7.62	−0.818 (−1.566, −0.071)	**0.032**

Control (1897)	Number (%)	952 (32.82)/808 (27.85)/137 (4.72)	—	—
	Age (years)	44.15 ± 14.81/43.30 ± 14.94/42.82 ± 13.69	—	—
	SBP (mm Hg)	115.06 ± 13.35/115.39 ± 13.84/114.07 ± 11.98	−0.223 (−1.15, 0.704)	0.637
	DBP (mm Hg)	74.10 ± 10.26/74.12 ± 9.95/73.63 ± 8.47	−0.205 (−0.907, 0.497)	0.566
	WC (cm)	80.50 ± 8.71/79.97 ± 8.64/79.63 ± 8.26	−0.519 (−1.098, 0.06)	0.079
	FBG (mg/dL)	92.67 ± 18.83/92.24 ± 16.87/90.55 ± 12.42	−0.822 (−2.067, 0.424)	0.196
	LogTG (mg/dL)	1.92 ± 0.19/1.95 ± 0.19/2.02 ± 0.18	0.042 (0.029, 0.055)	2.158**E** − 10
	HDL-C (mg/dL)	52.01 ± 12.78/50.75 ± 12.30/48.55 ± 11.67	−1.304 (−2.174, −0.435)	**0.003**

Values are indicated as the mean ± standard deviation.

*P* value: multiple regression analysis adjusted for age and sex (TT, TC, CC).

Abbreviations: CI: confidence interval; SBP: systolic blood pressure; DBP: diastolic blood pressure; WC: waist circumference; FBG: fasting blood glucose; LogTG: log-transformed triglyceride; HDL-C: high-density lipoprotein cholesterol.

Bold indicates statistical significance (*P* < 0.05).

**Table 3:** The prevalence of MS risk factors according to *APOA5* −1131 genotype.

	Variables	Number (%)				*P* value
		TT (1404)	TC (1237)	CC (260)	Total (2901)
Total (2901)	High FBG	423 (30.13)	398 (32.17)	96 (36.92)	917 (31.61)	0.082
	Low HDL-C	674 (48.01)	639 (51.66)	156 (60.00)	1469 (50.64)	**0.001**
	High BP	448 (31.91)	411 (33.23)	110 (42.31)	969 (33.40)	**0.005**
	High TG	312 (22.22)	345 (27.89)	106 (40.77)	763 (26.30)	8.637**E** − 10
	Large WC	720 (51.28)	604 (48.83)	135 (51.92)	1459 (50.29)	0.389

Male (1040)	High FBG	178 (37.55)	155 (33.55)	44 (42.31)	377 (36.25)	0.142
	Low HDL-C	177 (37.34)	204 (44.16)	52 (50.00)	433 (41.63)	**0.001**
	High BP	181 (38.19)	189 (40.91)	46 (44.23)	416 (40.00)	0.059
	High TG	141 (29.75)	158 (34.20)	49 (47.12)	348 (33.46)	1.718**E** − 04
	Large WC	170 (35.86)	157 (33.98)	44 (42.31)	371 (35.67)	0.101

Female (1861)	High FBG	245 (26.34)	243 (31.35)	52 (33.33)	540 (29.02)	0.294
	Low HDL-C	497 (53.44)	435 (56.13)	104 (66.67)	1036 (55.67)	0.316
	High BP	267 (28.71)	222 (28.65)	64 (41.03)	553 (29.72)	**0.045**
	High TG	171 (18.39)	187 (24.13)	57 (36.54)	415 (22.30)	1.146**E** − 04
	Large WC	550 (59.14)	447 (57.68)	91 (58.33)	1088 (58.46)	0.187

Values are indicated as number (%).

*P* value: test for difference of proportions (TT, TC, CC).

Abbreviations: MS: metabolic syndrome; FBG: fasting blood glucose; HDL-C: high-density lipoprotein cholesterol; TG: triglyceride; WC: waist circumference.

Bold indicates statistical significance (*P* < 0.05).

**Table 4:** Odds ratio of MS according to carriers of the minor allele of the *APOA5* −1131T>C polymorphism.

	Subjects	MAF	TT/TC/CC	OR (95% CI)	*P* value
Total (2901)	MS	0.336	452/429/123	1.322 (1.165, 1.501)	1.48**E** − 05
	Control	0.285	952/808/137	—	
Male (1040)	MS	0.352	151/154/47	1.285 (1.050, 1.574)	**0.015**
	Control	0.307	323/308/57	—	
Female (1861)	MS	0.327	301/275/76	1.339 (1.138, 1.576)	4.52**E** − 04
	Control	0.273	629/500/80	—	

Abbreviations: MS: metabolic syndrome; MAF: minor allele frequency; OR: odds ratio; CI: confidence interval.

Subjects: MS: subjects with ≥3 MS risk factors; control: subjects with <3 MS risk factors.

*P* value: adjusted for age and/or sex in multiple logistic regression analysis in an additive model.

Bold indicates statistical significance (*P* < 0.05).
