# Supplementary material for: Association of Apolipoprotein A5 Gene −1131T>C Polymorphism with the Risk of Metabolic Syndrome in Korean Subjects
Source: Biomed Res Int. 2013 Jan 28;2013:585134. doi: 10.1155/2013/585134 (PMC3581288; doi:10.1155/2013/585134)
Supplement: Supplementary file 1 — Supplementary Table 1. General characteristics of the study subjects according to gender. Supplementary Table 2. Association of the APOA5 -1131T>C polymorphism with MS parameters according to gender. [file 585134.f1.doc]

Supplementary Table 1. General characteristics of the study subjects according to gender

| Variables | Total | | MS | | Control | | MS Male and  Control Male  (*P*-value) | MS Female and  Control Female (*P*-value) |
| --- | --- | --- | --- | --- | --- | --- | --- | --- |
|  | Male(1040) | Female(1861) | Male(352) | Female(652) | Male(688) | Female(1029) |  |  |
| Age (years) | 46.98±15.87 | 48.20±15.20 | 53.11±13.70 | 56.72±13.48 | 43.84±16.00 | 43.61±14.06 | **2.706E-21** | **5.885E-77** |
| SBP (mmHg) | 123.13±15.09 | 118.11±15.70 | 130.75±15.73 | 127.96±15.37 | 119.23±13.15 | 112.80±13.09 | **4.617E-29** | **1.501E-85** |
| DBP (mmHg) | 79.04±10.93 | 75.93±11.14 | 83.63±11.26 | 82.13±10.95 | 76.70±9.98 | 72.58±9.71 | **5.015E-21** | **1.664E-68** |
| WC (cm) | 86.23±9.00 | 82.61±9.93 | 92.73±6.79 | 89.89±8.02 | 82.91±8.14 | 78.68±8.56 | **1.945E-76** | **2.460E-140** |
| FBG (mg/dL) | 101.61±29.26 | 97.63±26.24 | 113.60±35.86 | 110.75±37.01 | 95.47±22.94 | 90.55±13.37 | **8.376E-17** | **3.588E-37** |
| LogTG (mg/dL) | 2.08±0.24 | 2.00±0.23 | 2.25±0.21 | 2.18±0.21 | 2.00±0.20 | 1.90±0.18 | **4.235E-67** | **9.989E-139** |
| HDL-C (mg/dL) | 43.35±11.03 | 49.45±12.84 | 36.39±7.60 | 41.61±8.57 | 46.92±10.82 | 53.67±12.78 | **1.059E-63** | **2.378E-112** |

Values are indicated as the mean ± standard deviation.

*P*-value: Student’s T-test result between MS and Control

Abbreviations: MS, metabolic syndrome; SBP, systolic blood pressure; DBP, diastolic blood pressure; WC, waist circumference; FBG, fasting blood glucose; LogTG, log transformed triglyceride; HDL-C, high-density lipoprotein cholesterol

Bold indicates statistical significance (*P* < 0.05).

Supplementary Table 2. Association of the *APOA5* −1131T>C polymorphism with MS parameters according to gender

|  | Variables | Male | | | Female | | |
| --- | --- | --- | --- | --- | --- | --- | --- |
|  |  | TT / TC / CC | Slope(95% CI) | *P*-value | TT / TC / CC | Slope(95% CI) | *P*-value |
| Total (2901) | Number (%) | 474(45.58) / 462(44.42) / 104(10.00) | ㅡ | ㅡ | 930(49.97) / 775(41.65) / 156(8.38) | ㅡ | ㅡ |
|  | Age  (years) | 47.72±16.04 / 45.95±16.08 / 48.23±13.89 | ㅡ | ㅡ | 48.04±15.28 / 48.16±15.14 / 49.37±15.09 | ㅡ | ㅡ |
|  | SBP  (mmHg) | 123.19±15.27 / 122.65±14.97 / 124.99±14.8 | 0.461(-0.916~1.837) | 0.511 | 117.88±15.73 / 117.92±15.68 / 120.46±15.45 | 0.600(-0.44~1.64) | 0.258 |
|  | DBP  (mmHg) | 78.78±11.37 / 79.02±10.80 / 80.30±9.36 | 0.617(-0.388~1.621) | 0.229 | 75.90±11.25 / 75.78±11.16 / 76.81±10.36 | 0.126(-0.638~0.889) | 0.747 |
|  | WC  (cm) | 86.34±8.80 / 85.84±9.29 / 87.49±8.50 | 0.251(-0.559~1.061) | 0.544 | 82.71±9.94 / 82.34±9.89 / 83.29±10.15 | -0.117(-0.747~0.514) | 0.715 |
|  | FBG  (mg/dL) | 101.89±28.97 / 101.41±28.82 / 101.17±32.61 | -0.190(-2.836~2.455) | 0.888 | 97.55±27.24 / 97.53±24.47 / 98.56±28.67 | 0.080(-1.715~1.875) | 0.930 |
|  | LogTG  (mg/dL) | 2.05±0.22 / 2.10±0.23 / 2.19±0.26 | 0.061(0.04~0.082) | **1.963E-08** | 1.97±0.23 / 2.01±0.23 / 2.11±0.23 | 0.053(0.038~0.068) | **3.772E-12** |
|  | HDL-C  (mg/dL) | 44.69±11.63 / 42.57±10.47 / 40.73±9.87 | -2.102(-3.099~-1.105) | **3.810E-05** | 50.20±13.03 / 49.15±12.85 / 46.45±11.10 | -1.444(-2.327~-0.561) | **0.001** |
| MS (1004) | Number (%) | 151(14.52) / 154(14.81) / 47(4.52) | ㅡ | ㅡ | 301(16.17) / 275(14.78) / 76(4.08) | ㅡ | ㅡ |
|  | Age  (years) | 54.15±13.99 / 52.16±13.78 / 52.91±12.45 | ㅡ | ㅡ | 56.76±13.88 / 56.47±13.41 / 57.44±12.26 | ㅡ | ㅡ |
|  | SBP  (mmHg) | 131.8±16.36 / 128.87±15.44 / 133.53±14.13 | -0.104(-2.502~2.295) | 0.932 | 128.16±15.84 / 127.15±15.49 / 130.08±12.84 | 0.314(-1.42~2.048) | 0.722 |
|  | DBP  (mmHg) | 83.19±12.44 / 83.47±10.49 / 85.55±9.60 | 0.928(-0.788~2.645) | 0.288 | 82.50±10.82 / 81.80±11.61 / 81.91±8.94 | -0.429(-1.673~0.815) | 0.498 |
|  | WC  (cm) | 92.63±6.23 / 92.83±7.36 / 92.70±6.67 | 0.027(-1.003~1.057) | 0.959 | 90.44±7.98 / 89.30±8.07 / 89.81±7.90 | -0.586(-1.486~0.313) | 0.201 |
|  | FBG  (mg/dL) | 113.46±34.85 / 114.05±34.53 / 112.55±43.37 | 0.389(-4.959~5.737) | 0.886 | 111.83±38.90 / 110.35±34.78 / 107.93±37.49 | -1.855(-6.023~2.313) | 0.382 |
|  | LogTG  (mg/dL) | 2.20±0.18 / 2.28±0.20 / 2.33±0.26 | 0.064(0.034~0.095) | **4.541E-05** | 2.16±0.20 / 2.19±0.22 / 2.23±0.22 | 0.035(0.011~0.059) | **0.004** |
|  | HDL-C  (mg/dL) | 37.21±8.28 / 35.59±6.74 / 36.38±7.82 | -0.747(-1.904~0.41) | 0.205 | 42.33±8.78 / 40.98±8.70 / 41.07±6.96 | -0.857(-1.828~0.115) | 0.084 |
| Control (1897) | Number (%) | 323(31.06) / 308(29.61) / 57(5.48) | ㅡ | ㅡ | 629(33.80) / 500(26.87) / 80(4.30) | ㅡ | ㅡ |
|  | Age  (years) | 44.71±16.08 / 42.84±16.27 / 44.37±13.93 | ㅡ | ㅡ | 43.86±14.13 / 43.59±14.07 / 41.71±13.49 | ㅡ | ㅡ |
|  | SBP  (mmHg) | 119.16±12.91 / 119.53±13.73 / 117.95±11.28 | -0.088(-1.629~1.452) | 0.910 | 112.96±13.10 / 112.85±13.29 / 111.31±11.76 | -0.307(-1.464~0.85) | 0.602 |
|  | DBP  (mmHg) | 76.73±10.21 / 76.80±10.27 / 75.96±6.57 | -0.121(-1.292~1.051) | 0.840 | 72.75±10.03 / 72.47±9.39 / 71.96±9.28 | -0.257(-1.135~0.621) | 0.566 |
|  | WC  (cm) | 83.39±8.27 / 82.35±8.12 / 83.19±7.39 | -0.410(-1.349~0.528) | 0.391 | 79.02±8.57 / 78.51±8.63 / 77.10±7.94 | -0.587(-1.318~0.144) | 0.116 |
|  | FBG  (mg/dL) | 96.48±23.98 / 95.09±23.08 / 91.79±14.56 | -1.747(-4.427~0.933) | 0.201 | 90.72±15.19 / 90.48±11.16 / 89.66±10.64 | -0.264(-1.467~0.939) | 0.667 |
|  | LogTG  (mg/dL) | 1.98±0.20 / 2.01±0.20 / 2.07±0.20 | 0.038(0.015~0.062) | **0.001** | 1.88±0.18 / 1.91±0.18 / 1.98±0.15 | 0.044(0.029~0.059) | **1.685E-08** |
|  | HDL-C  (mg/dL) | 48.19±11.33 / 46.06±10.27 / 44.32±10.00 | -2.092(-3.353~-0.831) | **0.001** | 53.97±13.05 / 53.64±12.56 / 51.56±11.88 | -0.829(-1.995~0.0336) | 0.163 |

Values are indicated as the mean ± standard deviation.

*P*-value: Multiple regression analysis adjusted for age (TT, TC, CC)

Abbreviations: CI, confidence interval; SBP, systolic blood pressure; DBP, diastolic blood pressure; WC, waist circumference; FBG, fasting blood glucose; LogTG, log transformed triglyceride; HDL-C, high-density lipoprotein cholesterol

Bold indicates statistical significance (*P* < 0.05).

Supplementary Figure 1.


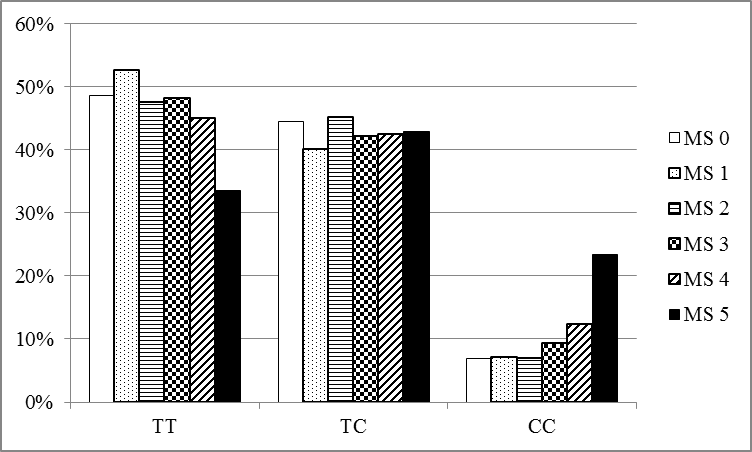


**Supplementary Figure legend**

Supplementary Figure 1. The percentage of subjects harboring one or more MS risk factors according to *APOA5* −1131 genotypes. The MS 0, MS 1, MS 2, MS 3, MS 4, and MS 5 indicate the number of MS risk factors within each subject.
